# Surface Feature Prediction for Laser Ablated 40Cr13 Stainless Steel Based on Extreme Learning Machine

**DOI:** 10.3390/ma16020505

**Published:** 2023-01-04

**Authors:** Zhenshuo Yin, Qiang Liu, Pengpeng Sun, Yinuo Zhou, Zhiwei Ning

**Affiliations:** 1 School of Mechanical Engineering and Automation, Beihang University, Beijing 100191, China; 2 Jiangxi Research Institute, Beihang University, Nanchang 330096, China; 3 Research and Application Center of Advanced CNC Machining Technology, Beijing 100191, China; 4 Research Center of High-Efficient and Green CNC Machining Process and Equipment, Beijing 100191, China

**Keywords:** laser ablation, 40Cr13 stainless steel, feature prediction, extreme learning machine, genetic algorithm

## Abstract

Determining an optimal combination of laser process parameters can significantly improve the efficiency and quality of 40Cr13 steel surface processing. In this study, two machine learning models (ELMSS and ELMPS) were proposed to predict the processing results of surface features to optimize process parameters. The prediction accuracies of the proposed models were always higher than those of traditional back propagation (BP) and radial basis function (RBF) neural networks, and the calculation time of the proposed models was significantly reduced. In comparison, the prediction accuracy ranking for ablation depth was ELMSS (92.6%), BP (89.8%), and RBF (89.6%), and for the ablation width, it was ELMSS (98.3%), BP (97.4%), and RBF (96.1%). The material removal rate was 92.4%, 91.1%, and 89.1% for ELMSS, BP, and RBF, respectively. Finally, the prediction accuracy ranking for surface roughness was 86.8%, 80.7%, and 79.5% for ELMPS, BP, and RBF, respectively. After optimization by the genetic algorithm, the prediction accuracies of the proposed models for the depth, width, material removal rate, and surface roughness reached 94.0%, 99.0%, 93.2%, and 91.2%, respectively. With the support of ELMSS and ELMPS, the results of the surface features can be predicted before machining and the appropriate process parameters can be selected in advance.

## 1. Introduction

Microstructures on the surface of 40Cr13 are widely used in the die industry owing to their properties, such as being aesthetically appealing, tribological, and easy to detach from injection points [1,2]. However, it is difficult to precisely process the ideal microstructure on the surface of 40Cr13 because of its hardness and strength. Laser ablation is a non-contact processing method that uses a focused spot of high energy density to instantly vaporize surface materials and is becoming an effective means of processing surface microstructures on hard-to-machine material [3,4]. The processing quality of laser ablation depends on the selection of process parameters, such as laser power, pulse frequency, and scanning speed [5]. Based on theoretical analysis and experimental results, choosing the right combination of process parameters can significantly improve the quality of laser ablation [6]. However, owing to the numerous combinations of process parameters in the laser ablation process, optimizing the process parameters through a trial-and-error approach can be time consuming. Therefore, finding a suitable method of improving optimal laser processing parameter selection to ultimately achieve precision laser ablation has become an urgent issue.

Machine learning has developed rapidly since it was proposed and has been successfully applied in the prediction of laser processing results [7]. Chen et al. [8] proposed a supervised machine learning approach to detect trajectory defects in selective laser melting, four metrics were taken as input variables to the model to quantitatively assess the quality of the trajectory. They believe that the machine learning model can significantly improve the search efficiency of process parameter windows and has great application potential in future unmanned factories. Yousef et al. [9] proposed a neural network that can be used to model a nonlinear laser micromachining process, which can help to predict the pulse energy level required to produce microstructures with the desired depth and diameter. Teixidor et al. [10] used three types of machine learning models to predict the process results of surface features. The inputs of these models are the scan speed, pulse intensity, and pulse frequency while the outputs are depth, width, surface roughness, and material removal rate (MRR). Jimin et al. [11] used a multi-layer neural network to predict the processing results of laser cutting. The inputs of the model included not only the typical parameters but also the slant angle of the laser head. Further, they applied the radial basis function (RBF) neural network to predict the processing results of stainless-steel sheets [12]. Dhupal et al. [13] applied artificial neural network (ANN) and response surface methodology to optimize the machining features of the pulsed Nd:YAG laser for microgrooving on aluminum titanate. The experimental results show that the prediction results of the two methods are consistent. Later, the genetic algorithm was applied to further adjust the model, which improved the prediction accuracy [14]. However, the calculation time of the algorithm was inevitably increased. Ciurana et al. [15] applied the ANN for predicting the quality of 3D geometrical features on AISI H13 steel and used the particle swarm optimization algorithm to improve the accuracy. The back propagation (BP) neural network, which is another typical prediction model, was successfully applied to optimize the process parameters of laser micro-machining [16,17].

Despite the successful application of the above machine learning models to the prediction of laser ablation surface features, a few limitations still exist. First, the above-mentioned models require a large amount of experimental data for training to achieve high prediction accuracy. In addition, the computational efficiency of these algorithms is relatively low and the training time of the models is on the second level, making them unsuitable for real-time applications.

The technological test of laser ablated 40Cr13 surfaces is typically time-consuming and laborious, and it is difficult to obtain a large amount of data to train a prediction model. Therefore, an accurate and efficient method that can be used to predict surface features with limited experimental data is required. To the best of our knowledge, there are only a few scientific studies on the laser ablation of 40Cr13 to obtain target surface features. Our objective is to bridge this knowledge gap and improve the laser ablation quality of superhard materials such as 40Cr13 stainless steel.

This paper presents an extreme learning machine of single-track scanning (ELMSS) and plane scanning (ELMPS) for the prediction of the surface features, which improve prediction accuracies and significantly reduce the training time.

The novelties of this study are that the extreme learning machine is applied to the prediction of the laser processing surface features for the first time, all the unknown parameters in the ELMSS and ELMPS are determined, and the accurate prediction of the surface feature of nanosecond laser processing 40Cr13 material is realized. The practical significance of the presented methodology is that researchers can use the proposed models to accurately predict the dimension of surface features according to the input laser processing process parameters. If the dimension cannot meet the tolerance requirements, the process parameters must be modified. In this way, In this way, trial and error experiments do not need to be conducted, which reduces the time and labor costs.

The rest of this paper is organized as follows: Section 2 introduces the experimental details. Section 3 presents the establishment of the prediction models. Section 4 compares the prediction accuracies and algorithm efficiency of presented models and those of two other ANN models. Section 5 utilizes the genetic algorithm to further improve the prediction accuracies of ELMSS and ELMPS. Finally, Section 6 presents the concluding remarks of the study.

## 2. Experiment Details

### 2.1. Material

40Cr13 stainless steel was used in this study, and its main components are listed in Table 1. The chemical composition analysis was conducted by suppliers (Yuzhixiang Material Co., Suzhou, China). 40Cr13 has high hardness, abrasion resistance, and corrosion resistance and is widely used to manufacture high-end precision molds. The surfaces of the samples were milled and ground before the technological tests to eliminate potential influences of the initial surface roughness on the machining results [17].

### 2.2. Experimental Platform

**Figure 1:**
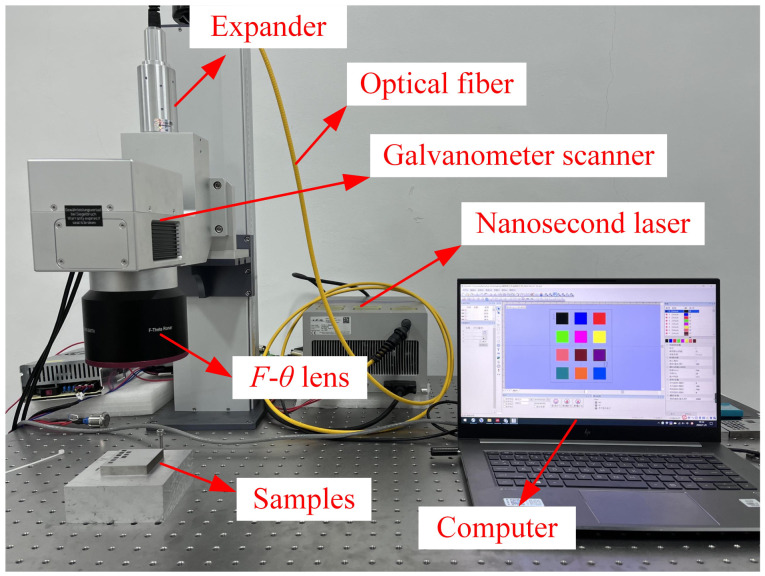
The experimental platform.

The experimental platform is shown in Figure 1. This study used a nanosecond pulse laser generator (FORMULA-355, Advanced Optowave Co., New York, NY, USA) with a wavelength of 355 nm, and its main parameters are listed in Table 2. The output spot of the laser generator enters a 3D galvanometer scanner (G3-3D, JCZ Technology Co., Beijing, China) with a focal length of 290 mm through a beam expander (BEX-355-6X, Wavelength OE Co., Nanjing, China) and uses an F-theta field lens (SL-355, Wavelength OE Co., Nanjing, China) to focus the laser spot on the surface of the samples. The diameter of focus spot is 19.38 μm. The laser power irradiating on the surfaces was measured with a power meter (Ophir 30A-SV-17, Thorlabs Co., Newton, MA, USA) [17].

### 2.3. Technological Test Methods

To obtain excellent properties, micro-grooves and planes are taken as the two typical microstructures on the surface of the dies. These two structures are usually obtained by single-track and plane scanning, respectively. The details of these methods are given below.

#### 2.3.1. Single-Track Scanning

**Figure 2:**
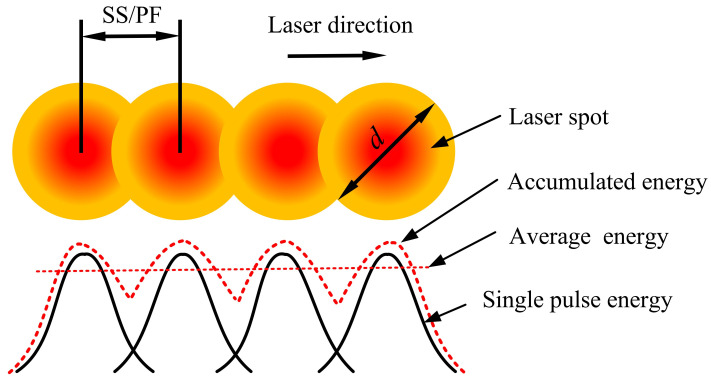
The laser pulse accumulation diagram of single-pass machining.

Single-track scanning is realized to ensure that the laser spot linearly scans the surface of a sample, producing a micro-groove structure by laser ablation. Its process parameters include laser power (LP), pulse frequency (PF), and scanning speed (SS). The laser pulse accumulation of single-pass machining is shown in Figure 2. Since the output power of a laser with a wavelength of 355 nm is significantly affected by the variation of the PF, the selection of the variation range of the process parameters must be reasonable. The process parameters of single-track scanning in this study are listed in Table 3. The value of a single process parameter is changed each time during processing, and other parameters remain unchanged. A total of 48 micro-grooves are processed [17].

#### 2.3.2. Plane Scanning

**Figure 3:**
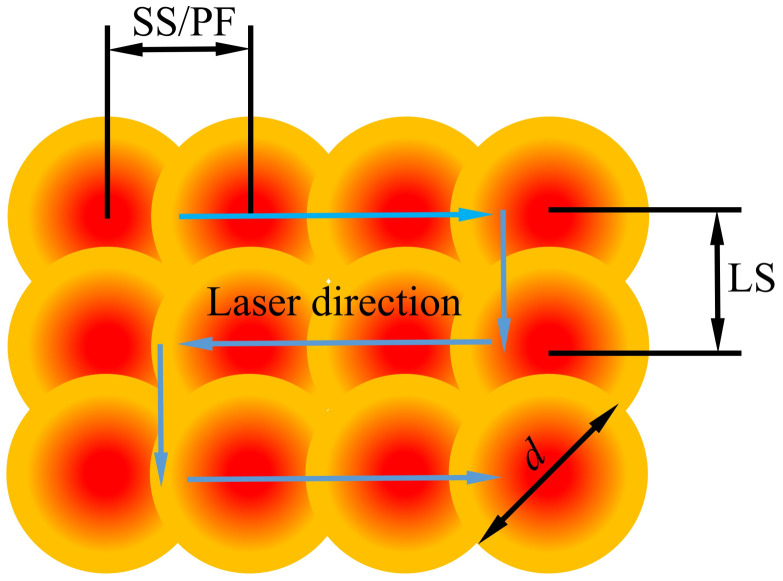
The laser pulse accumulation diagram of plane scanning.

Plane scanning is realized using the laser spot for reciprocating the scanning motion on the surface of a sample with a certain line spacing (LS). Its process parameters add the LS based on single-track scanning parameters. The laser pulse accumulation of plane scanning is shown in Figure 3. The variation range of the process parameters is listed in Table 4. Similarly, the value of a single process parameter is changed during each processing while others remain fixed. A total of 81 planes are formed.

### 2.4. Features Measurement

**Figure 4:**
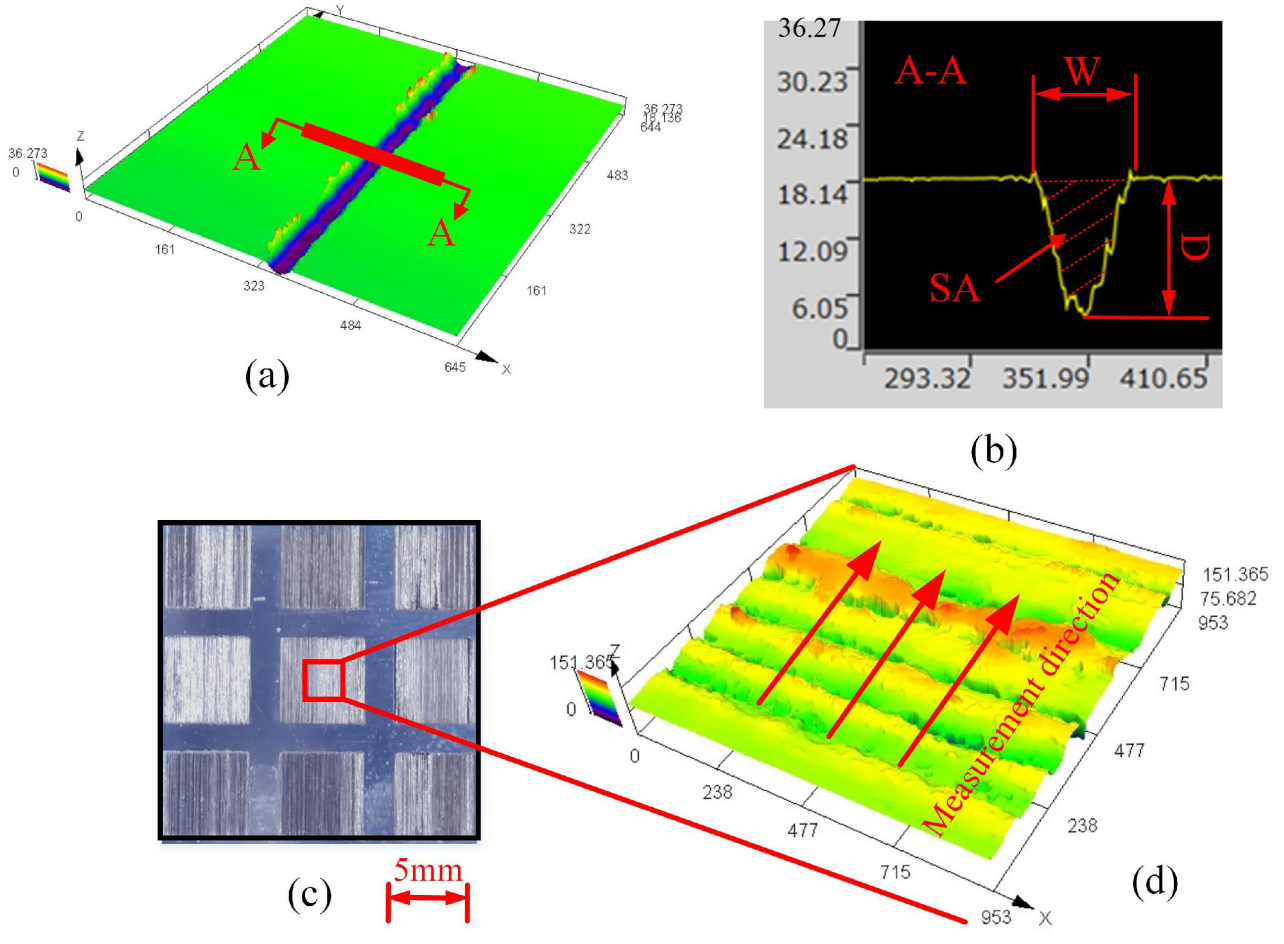
Schematic of machining results and measurement process. (**a**) Typical micro-groove diagram observed by LEXT OLS5100. (**b**) Section profile of the micro-groove. (**c**) Physical image of the rough surface after plane scanning. (**d**) Typical surface morphology observed by LEXT OLS5100.

The cross-section of the microgroove structure produced by single-track laser ablation is approximately V-shaped owing to the Gaussian distribution of the laser beam [18]. The micro-groove of single-track scanning was measured by laser confocal microscopy (LEXT OLS5100, Olympus Co., Tokyo, Japan), and a typical micro-groove is shown in Figure 4a where the cross-section of the micro-groove is observed along the A-A direction. As is shown in Figure 4b, three features are defined on the cross-section: depth (D), width (W), and sectional area (SA). The material removal rate (MRR) is defined as

(1) MRR=SA×SS×t

where SA denotes the sectional area of the micro-groove, SS denotes the scanning speed, and *t* denotes the scanning time.

During the measurement of D, W, and MRR, three sections with a length of 1 mm were cut along each micro-groove, and the average value was taken as the measurement result of the three features.

The physical image of the surface obtained from the plane scanning is shown in Figure 4c, and a typical surface morphology observed using LEXT OLS5100 is exemplified in Figure 4d. A contact roughness meter (SJ-310, Mitutoyo Co., Kawasaki, Japan) was used to measure the surface roughness (Ra) along the direction shown in Figure 4d. Ra was calculated as the average value of three measurements.

Measurement results of the four surface features—D, W, MRR, and Ra—obtained using the above mentioned methods are shown in Table A1 of Appendix A, which contains 48 samples of single-track scanning and 81 samples of plane scanning.

## 3. Establishment of the Prediction Model

**Figure 5:**
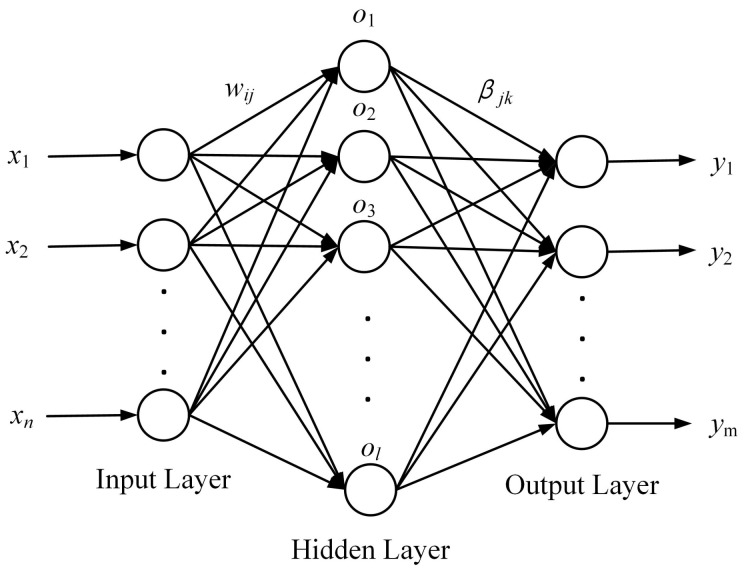
Typical single hidden layer neural network structure.

ELM was proposed by Guang-Bin Huang et al. [19] in 2004 and was soon applied to function fitting and classification problems [20,21,22]. It is part of a single-hidden-layer feedforward neural network. A typical structure of ELM is shown in Figure 5, consisting of input, hidden, and output layers. Assuming that n,l,m were the neuron numbers of the input, hidden, and output layers, respectively, the connection weight matrix between the input and hidden layers is

(2) $$W={\left[\begin{array}{cccc} w_{11} & w_{12} & \cdots & w_{1n}\\ w_{21} & w_{22} & \cdots & w_{2n}\\ \cdots & \cdots & \cdots & \cdots \\ w_{l1} & w_{l2} & \cdots & w_{ln} \end{array}\right]}_{l\times n}$$

where *w*${}_{ji}$ represents the connection weight between the *i*${}_{th}$ neuron in the input layer and *j*${}_{th}$ neuron in the hidden layer.

The connection threshold matrix of the hidden layer is

(3) $$b={\left[\begin{array}{cccc} b_{1} & b_{2} & \cdots & b_{l} \end{array}\right]}^{T}$$

where *b*${}_{i}$ represents the connection threshold of the *i*${}_{th}$ neuron in the hidden layer.

The connection weight matrix between the hidden and output layers is

(4) $$\beta ={\left[\begin{array}{cccc} \beta_{11} & \beta_{12} & \cdots & \beta_{1m}\\ \beta_{21} & \beta_{22} & \cdots & \beta_{2m}\\ \cdots & \cdots & \cdots & \cdots \\ \beta_{l1} & \beta_{l2} & \cdots & \beta_{lm} \end{array}\right]}_{l\times m}$$

where β${}_{jk}$ represents the connection weight between the *j*${}_{th}$ neuron in the hidden layer and *k*${}_{th}$ neuron in the output layer.

Assuming that there are *P* samples in the training set data, the input and output matrices of ELM can be expressed, respectively, as

(5) $$X={\left[\begin{array}{cccc} x_{11} & x_{12} & \cdots & x_{1P}\\ x_{21} & x_{22} & \cdots & x_{2P}\\ \cdots & \cdots & \cdots & \cdots \\ x_{n1} & x_{n2} & \cdots & x_{nP} \end{array}\right]}_{n\times P}$$

(6) $$Y={\left[\begin{array}{cccc} y_{11} & y_{12} & \cdots & y_{1P}\\ y_{21} & y_{22} & \cdots & y_{2P}\\ \cdots & \cdots & \cdots & \cdots \\ y_{m1} & y_{m2} & \cdots & y_{mP} \end{array}\right]}_{m\times P}$$

If *g*(*x*) denotes the activation function, the output matrix of the hidden layer then becomes

(7) $$H={\left[\begin{array}{cccc} g(w_{1}x_{1}+b_{1}) & g(w_{2}x_{1}+b_{2}) & \cdots & g(w_{l}x_{1}+b_{l})\\ g(w_{1}x_{2}+b_{1}) & g(w_{2}x_{2}+b_{2}) & \cdots & g(w_{l}x_{2}+b_{l})\\ \cdots & \cdots & \cdots & \cdots \\ g(w_{1}x_{P}+b_{1}) & g(w_{2}x_{P}+b_{2}) & \cdots & g(w_{l}x_{P}+b_{l}) \end{array}\right]}_{P\times l}$$

Guang-Bin Huang et al. [19] pointed out that, when the activation function *g*(*x*) is infinitely differentiable, the connection weight between the hidden and output layers can be obtained using:

(8) $$\beta =H^{+}Y$$

where *H*${}^{+}$ is the Moore–Penrose inverse of the hidden layer output matrix.

The connection weight (*W*) and threshold (*b*) between the input and hidden layers can be randomly generated before training and remain unchanged during the training process. After the activation function *g*(*x*) is determined before training and the connection weight (β) is solved using Equation (8), all the parameters of ELM are determined. The entire training process does not require error back-propagation and iterative calculation. Hence, the training time of ELM can be greatly reduced on the premise of ensuring prediction accuracy.

Assuming that there are *Q* samples in the test set data, the prediction result of ELM is:

(9) $$T={\left[\begin{array}{cccc} T_{1} & T_{2} & \cdots & T_{Q} \end{array}\right]}_{m\times Q}$$

(10) $$T_{j}=\left[\begin{array}{c} t_{1j}\\ t_{2j}\\ \cdots \\ t_{mj} \end{array}\right]=\left[\begin{array}{c} \sum_{i=1}^{l}\beta_{i1}g\left(w_{i}x_{j}+b_{i}\right)\\ \sum_{i=1}^{l}\beta_{i2}g\left(w_{i}x_{j}+b_{i}\right)\\ \cdots \\ \sum_{i=1}^{l}\beta_{im}g\left(w_{i}x_{j}+b_{i}\right) \end{array}\right]\left(j=1,2,...Q\right)$$

where *w*${}_{i}$ and *b*${}_{i}$ are the preset connection weights and thresholds, respectively, β${}_{i}$ is the calculation result of training, and *x*${}_{i}$ is the input data of the test set.

**Figure 6:**
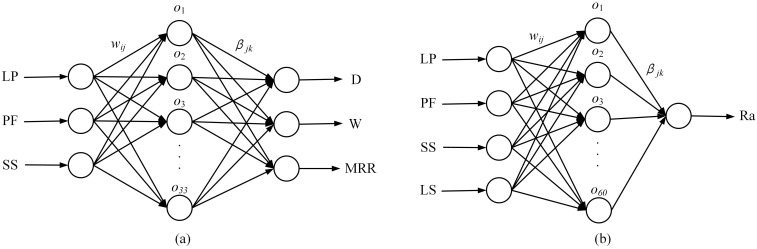
Prediction models for surface features based on ELM. (**a**) ELMSS model for feature prediction. (**b**) ELMPS model for feature prediction.

The ELM model for predicting the surface features was developed using MATLAB R2018a software. The single-track scanning (ELMSS) model proposed for feature prediction is shown in Figure 6a. LP, PF, and SS were set as the input layer parameters, and D, W, and MRR as the output layer parameters. The number of neurons in the hidden layer was 33, and the activation function was “sigmoid.” The plane scanning (ELMPS) model is shown in Figure 6b. LS was added to the input layer parameters, and the output layer parameter was changed to Ra. The number of neurons in the hidden layer was 60, and the activation function was again “sigmoid”.

Groups 1–45 of data in Table A1 of Appendix A were treated as the training set and groups 46–48 as the test set. Based on the ELM model, the surface features were predicted through the following process.

First, the technological test was carried out according to the designed parameters, and the machining result data of the surface features were obtained.

Second, the data were categorized into training and test sets after they were normalized, and the number of neurons in the hidden layer was determined. The connection weights (*W*) and thresholds (*b*) between the input layer and the hidden layer were randomly set.

Third, an infinitely differentiable activation function was selected, and the hidden layer output matrix (*H*) combining the data of the training sets was calculated.

Fourth, the connection weights (β) between the hidden and output layers were calculated to complete the training of the ELM model.

Finally, the test set data were inputted into the trained model, the predicted results were compared with the measured results, and the prediction accuracy of the ELMSS and ELMPS was analyzed.

The ELMSS and ELMPS are based on a typical perceptron with one hidden layer. The innovation of this paper is to find the most suitable number of neurons in the hidden layer used to predict the surface feature of laser processing and use the genetic algorithm to optimize the connection weight and threshold to obtain the highest prediction accuracy.

## 4. Prediction Results of ELMSS and ELMPS

To verify the accuracy of the ELMSS, the prediction results were compared with that of BP [16] and RBF [12] neural network machine learning models for the same training and test data sets. For the prediction models of single-track scanning, the network structure of the BP neural network is 3-6-3, which is the network structure with the highest prediction accuracy. The activation function of BP neural network is “tansig,” which is infinitely differentiable. The expression of the function is:

(11) $$tansig\left(x\right)=\frac{2}{1-e^{-2x}}-1$$

The spread value of the RBF neural network is set to 1.0. The measured values and predicted results of D, W, and MRR after training with the same data are shown in Table 5.

The average prediction accuracy is defined as

(12) $$\varepsilon =\left(1-\frac{\sum_{1}^{N}\left(\frac{\left|X_{i}-\mu \right|}{\mu }\right)}{N}\right)\times 100\%$$

From the calculation for prediction accuracy, ELMSS has the highest accuracy for D, reaching 92.6%, followed by that of the BP and RBF neural networks, which are 89.8% and 89.6%, respectively. The prediction accuracies of the three models for W are relatively high. Among them, the ELMSS has the highest prediction accuracy of 98.3%, followed by the BP and RBF neural networks, with prediction accuracies of 97.4% and 96.1%, respectively. The prediction accuracy ranking for MRR is ELMSS (92.4%), BP neural network (91.1%), and RBF neural network (89.1%), respectively.

Similarly, to verify the accuracy of the ELMPS, the prediction results were compared with two other models, the network structure of the BP neural network is 4-3-1, the activation function is “tansig,” and the spread value of the RBF is still set to 1.0. The measured values and prediction results of Ra by the three models are shown in Table 6.

The average prediction accuracy of plane scanning Ra was also calculated. The average prediction accuracies of the three models for Ra were relatively low. ELMPS had the highest average accuracy of 86.8%, and the average prediction accuracies of the BP and RBF neural networks were 80.7% and 79.5%, respectively.

**Figure 7:**
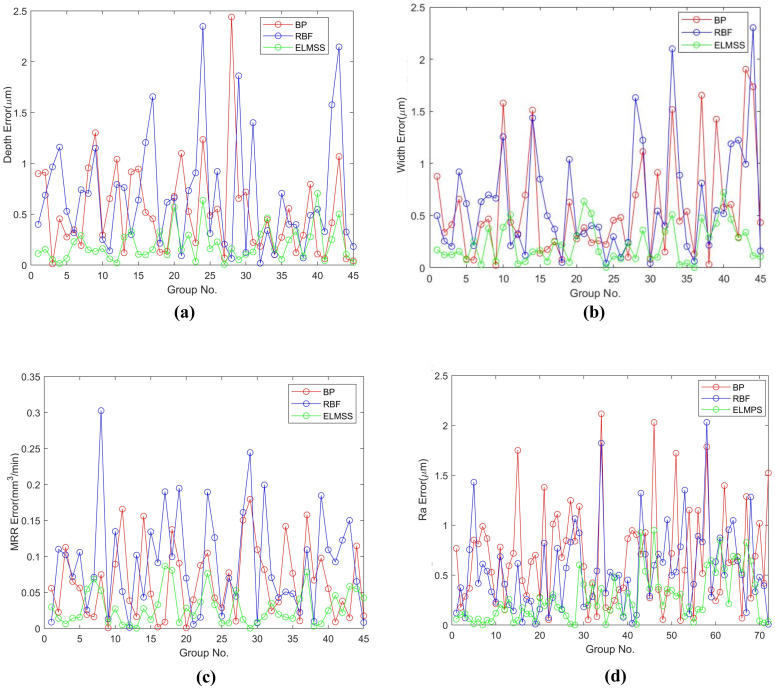
Absolute error between the predicted value and actual value of the three types of models. (**a**) Absolute error of D of three types of models. (**b**) Absolute error of W of three types of models. (**c**) Absolute error of MRR of three types of models. (**d**) Absolute error of Ra of three types of models.

To test the generalization performance of ELMSS and ELMPS, 45 samples of the training set data in single-track scanning were treated as the input of the models, and 45 predicted values of D, W, and MRR were obtained. Further, the absolute error between the predicted and actual values was plotted in Figure 7a–c. Then, 72 samples of the training set data in plane scanning were used as the input of the models, and 72 predicted values of Ra were obtained. The absolute error between the predicted and actual values is plotted in Figure 7d. Figure 7 shows that the absolute error of ELMSS and ELMPS is minimal compared to the other two models.

The training period of the models were used to compare the computing efficiency of the three models. The CPU performance of the computing platform was unified as Intel(R) Core(TM) i7-10750H CPU @ 2.60GHz. The values of the training time are listed in Table 7. The BP neural network requires the longest calculation time, because the training process of the model needs error back propagation and iteration. The RBF neural network belongs to the forward network, but the calculation time is longer than that of ELMSS and ELMPS. In summary, the computational efficiency of the prediction models proposed in this study have been improved from tens to hundreds of times compared with the other two types of machine learning models, providing the possibility of using the ELMSS and ELMPS in real-time applications.

## 5. Optimization of ELMSS and ELMPS Based on Genetic Algorithm

The connection weights and thresholds from the input layer to the hidden layer of the ELMSS and ELMPS models are randomly determined, which leads to unstable prediction results. This section applied genetic algorithm to optimize the parameters of prediction models, in order to obtain the optimal connection weights and thresholds, thereby improving the prediction accuracy of the models.

**Figure 8:**
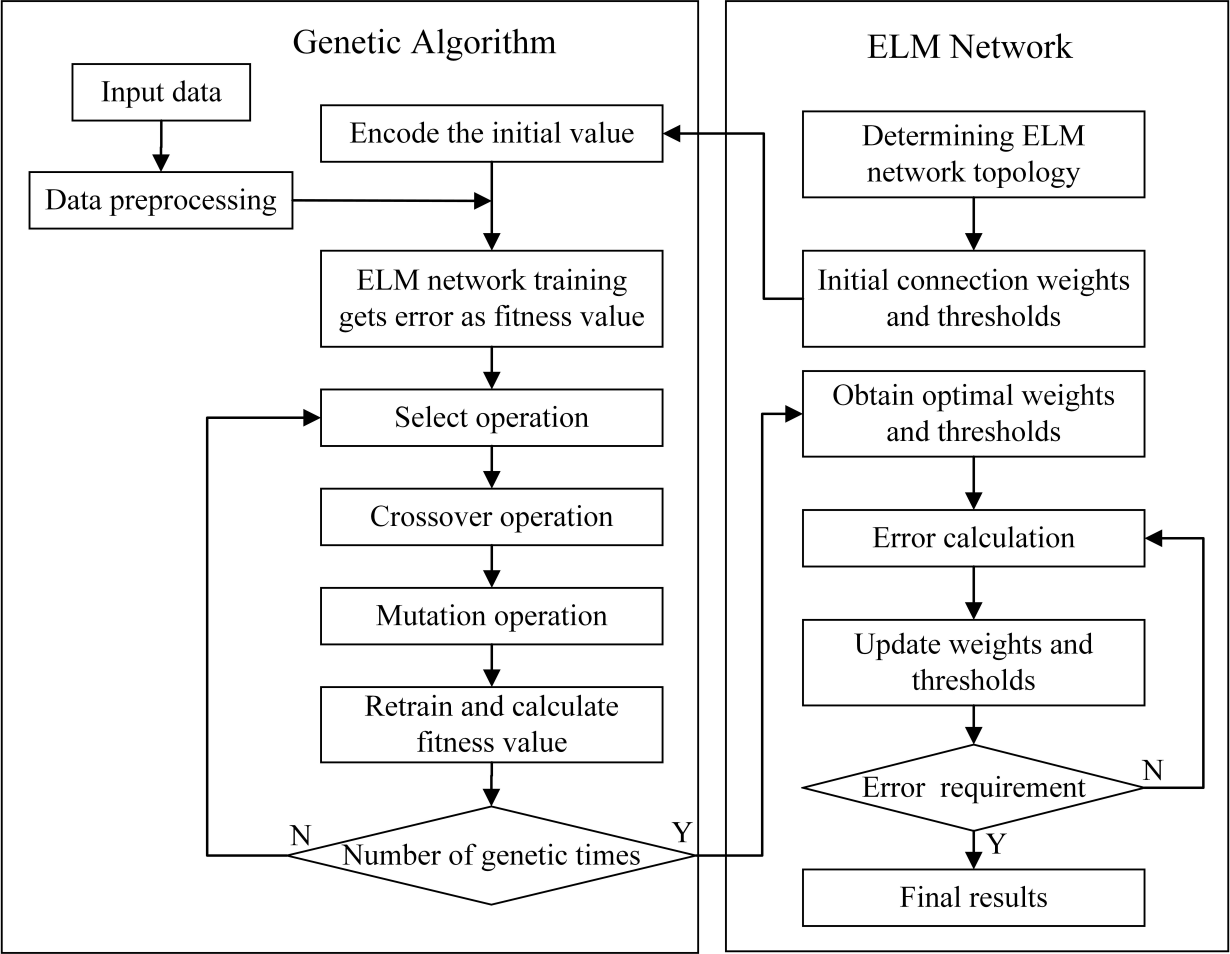
The flowchart of genetic algorithm optimized prediction models.

When optimizing the ELMSS and ELMPS models, the genetic algorithm first encodes the connection weights and thresholds of the model to form a certain number of individuals, and then sets the solutions that may have optimal solution individuals to form the population to be optimized. After the population is formed, individuals are screened through selection, crossover, and mutation operations according to the selected fitness function to complete a generation of evolution. Each generation retains the individual with the best fitness value, and generation-by-generation evolution produces approximate solutions with smaller and smaller errors. The flowchart of genetic-algorithm-optimized prediction models is shown in Figure 8.

When performing a selection operation, the fitness function of the individual should be calculated first, and the calculation method is

(13) $$F=\sqrt{\frac{1}{N}\sum_{1}^{N}{\left(X_{i}-\mu \right)}^{2}}$$

Among them, *X*${}_{i}$ is the predicted value of the sample, μ is the actual value of the sample, and *N* is the number of samples in the test set.

Then, the probability that an individual is selected to remain in the next generation is

(14) $$f_{i}=1/F_{i}$$

(15) $$p_{i}=f_{i}/\sum_{j=1}^{N}f_{j}$$

The crossover operation is to select a part of variables in two individuals to exchange at the same chromosome position. The *k*${}_{th}$ individual *a*${}_{k}$ and the *l*${}_{th}$ individual *a*${}_{l}$ crossover at position *j* as follows:

(16) $$\left\{\begin{array}{c} a_{kj}=a_{kj}\left(1-b\right)+a_{lj}b\\ a_{lj}=a_{lj}\left(1-b\right)+a_{kj}b \end{array}\right.$$

Among them, *b* is a random number between 0 and 1, and the probability of crossover between each generation is 0.7.

The mutation operation is that an individual itself mutates at a certain chromosome position. The mutation operation of the *i*${}_{th}$ individual at position *j* is as follows

(17) $$f\left(g\right)={\left(1-g/G_{\max}\right)}^{2}$$

(18) $$a_{ij}=\left\{\begin{array}{c} a_{ij}+\left(a_{ij}-a_{\max}\right)\times f\left(g\right)\;r>0.5\\ a_{ij}+\left(a_{\min}-a_{ij}\right)\times f\left(g\right)\;r\leq 0.5 \end{array}\right.$$

Among them, *g* is the current evolutionary generation, *G*${}_{max}$ is the maximum evolutionary generation, *a*${}_{max}$ and *a*${}_{min}$ are the maximum and minimum values of variables in the individual, respectively, and *r* is a random number between 0 and 1, and the probability of mutation between each generation is 0.01.

**Figure 9:**
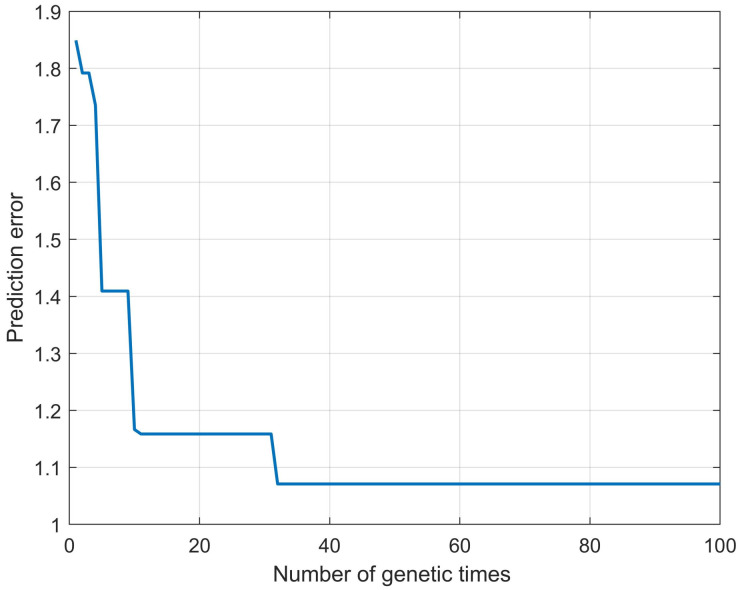
The change process of prediction errors of GA-ELMSS.

In ELMSS optimized by genetic algorithm (GA-ELMSS), the maximum number of genetic iterations is set to 100, and the change process of prediction errors are shown in Figure 9.

**Figure 10:**
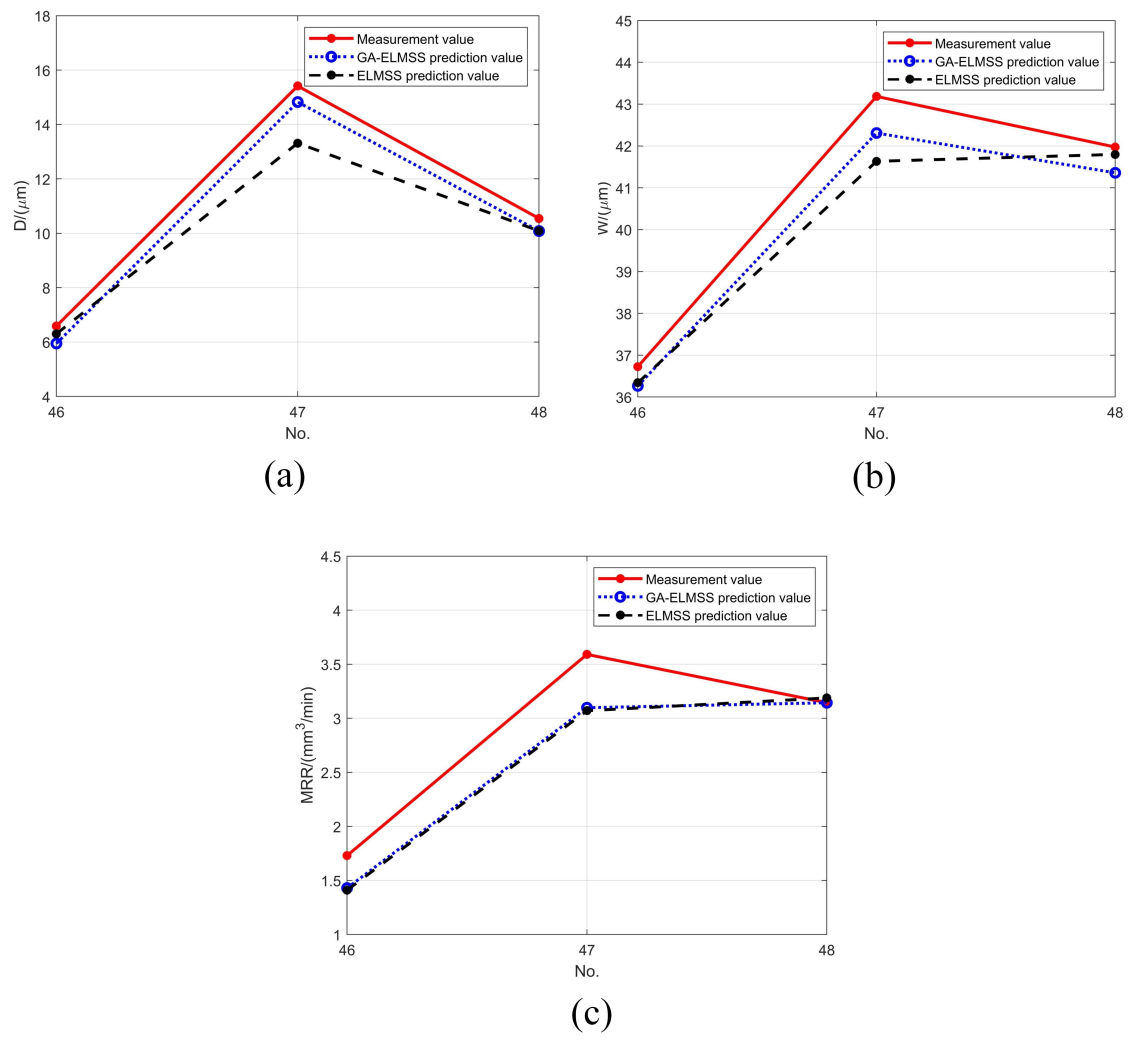
Comparison of the prediction results of the GA-ELMSS. (**a**) The prediction results of GA-ELMSS for D. (**b**) The prediction results of the GA-ELMSS for W. (**c**) The prediction results of GA-ELMSS for MRR.

The connection weights and thresholds of GA-ELMSS are shown in Table A2 of Appendix A. At this point, all of the parameters of the ELMSS have been determined, which can be directly applied to the prediction of single-pass machining. Groups 1–45 of data in Table A1 of Appendix A are treated as the training set, and groups 46–48 as the test set. The comparison of the prediction results of the ELMSS before and after the optimization of the genetic algorithm on D, W and MRR are shown in Figure 10.

The prediction accuracies are calculated according to Equation (12), and the results are shown in Table 8. It was found that the genetic algorithm can further improve the prediction accuracies of the GA-ELMSS.

**Figure 11:**
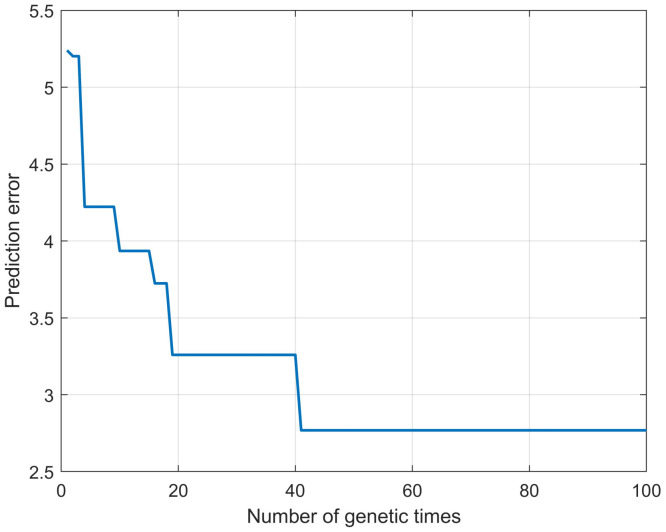
The change process of prediction errors of GA-ELMPS.

In ELMPS optimized by genetic algorithm (GA-ELMPS), the maximum number of genetic iterations is also set to 100, and the change process of prediction errors are shown in Figure 11.

**Figure 12:**
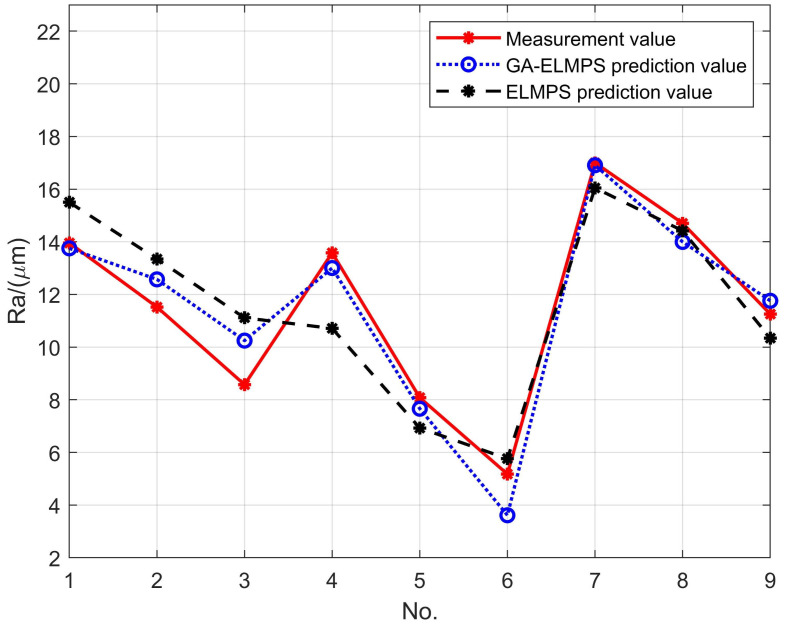
The prediction results of the GA-ELMPS on Ra.

The connection weights and thresholds of GA-ELMSPS are shown in Table A3 of Appendix A. Similarly, groups 1–45 of data in Table A1 of Appendix A are treated as the training set, and groups 46–48 as the test set. The comparison of the prediction results of the ELMPS before and after the optimization of the genetic algorithm on Ra are shown in Figure 12.

The prediction accuracy is also calculated according to Equation (12), and the results are shown in in Table 9. It was found that the genetic algorithm improves the prediction accuracy of the ELMPS model, with greater improvement than that of ELMSS.

## 6. Conclusions

Surface feature prediction models for laser ablated 40Cr13 stainless steel based on ELM are proposed in this paper. The results of D, W, and MRR processed by single-track scanning were predicted by the ELMSS model, and the results of Ra processed by plane scanning were predicted by the ELMPS model. The prediction accuracy and algorithm efficiency of the proposed models are superior to the BP [16] and RBF [12] neural networks. The main conclusions are summarized as follows:

1. The ELMSS model has the highest prediction accuracy for the W, with an average prediction accuracy of 98.3%, followed by D and MRR, which are 92.6% and 92.4%, respectively. Meanwhile, the prediction accuracy for Ra of ELMPS is relatively low, with an average prediction accuracy of 86.8%. Therefore, the models proposed in this paper are relatively more suitable for predicting surface feature of single-pass scanning.
2. The prediction accuracies of the ELMSS and ELMPS are higher than that of the BP and RBF neural networks. The prediction accuracies for D were 92.6%, 89.8%, and 89.6% for the ELMSS, BP, and RBF neural networks, respectively; they were 98.3%, 97.4%, and 96.1% for W, respectively; they were 92.4%, 91.1%, and 89.1% for MRR, respectively. The prediction accuracies for Ra were 86.8%, 80.7%, and 79.5% for the ELMPS, BP, and RBF neural networks, respectively. In general, the prediction accuracies of the models proposed in this paper are higher than those of the above two traditional ANN models.
3. The computation time of the ELMSS and ELMPS model is significantly lower than that of the other two machine learning methods. In terms of computational efficiency, the models can be ordered as ELMPS, ELMSS, RBF, and BP neural network. Moreover, the computation time of the model proposed in this paper is at the millisecond level, which can be used for the real-time control of laser processing scenarios.
4. Through genetic algorithm optimization, the prediction accuracies of ELMSS and ELMPS are further improved. The prediction accuracies of D, W, and MRR are improved from 92.6%, 98.3%, and 92.4% to 94.0%, 99.0%, and 93.2%, respectively. The prediction accuracy of Ra is improved from 86.8% to 91.2%.

In this study, ELMSS and ELMPS were used for the first time in the field of laser processing to accurately predict the features of the ablated surface. With the support of these two models, the results of surface features can be predicted in advance before processing, allowing appropriate process parameters to be selected. Operators do not need much experience or time for experimentation to produce a desirable product. Further, the algorithms of ELMSS and ELMPS are extremely efficient, and determining how to apply them to real-time control will be studied in the future.

## Figures and Tables

**Table 1:** Main components of 40Cr13 stainless steel (wt%).

C	Si	Mn	S	P	Cr	Ni
0.36–0.45	≤0.60	≤0.80	≤0.03	≤0.035	12.00–14.00	≤0.60

**Table 2:** Main parameters of laser generator.

Wavelength	Maximum Power	M^2^ Factor	Spot Diameter	Pulse Width	Repetition Rate
355 nm	25 W	1.2	19.38 μm	<20 ns	40–300 kHz

**Table 3:** Process parameters of single-track scanning.

Process Parameters	Range of Values			
LP/(W)	10	14	18	
PF/(kHz)	40	50	60	70
SS/(mm/s)	100	150	200	250

**Table 4:** Process parameters of plane scanning.

Process Parameters	Range of Values		
LP/(W)	10	14	18
PF/(kHz)	50	60	70
SS/(mm/s)	100	150	200
LS/(μm)	5	10	15

**Table 5:** Measured value and prediction results of the three types of models.

No.	D/(μm)				W/(μm)				MRR/(mm^3^/min)			
	BP	RBF	ELMSS	Exp.	BP	RBF	ELMSS	Exp.	BP	RBF	ELMSS	Exp.
46	6.222	7.140	6.302	6.590	37.754	36.617	36.727	36.341	1.627	1.465	1.611	1.730
47	11.718	12.924	13.313	15.415	41.407	43.985	43.184	41.633	2.996	3.137	3.070	3.591
48	10.447	11.256	10.087	10.545	43.236	39.542	41.974	41.798	3.280	3.290	3.188	3.144

**Table 6:** Measured values and prediction results of Ra.

No.	Ra/(μm)			
	BP	RBF	ELMPS	Exp.
46-1	14.735	15.804	15.500	13.956
46-2	12.886	12.819	13.347	11.522
46-3	10.754	11.779	11.112	8.573
47-1	12.497	11.552	10.709	13.578
47-2	9.771	9.427	6.927	8.079
47-3	6.860	5.646	5.762	5.176
48-1	13.802	12.968	16.045	16.999
48-2	11.149	10.843	14.419	14.704
48-3	8.322	7.675	10.342	11.253

**Table 7:** Training time of the three types of models.

	BP	RBF	ELMSS	ELMPS
Single-track scanning	1.896 s	0.147 s	0.005 s	-
Plane scanning	1.208 s	0.211 s	-	0.002 s

**Table 8:** Prediction accuracies of ELMSS and GA-ELMSS.

	ELMSS	GA-ELMSS
D	92.6%	94.0%
W	98.3%	99.0%
MRR	92.4%	93.2%

**Table 9:** Prediction accuracies of ELMPS and GA-ELMPS.

	ELMPS	GA-ELMPS
Ra	86.8%	91.2%

## Data Availability

The research data was provided in Appendix A.

## Abbreviations

The following abbreviations are used in this manuscript:

ELMSS	Extreme learning machine of single-track scanning
ELMPS	Extreme learning machine of plane scanning
MRR	Material removal rate
BP	Back propagation
RBF	Radial basis function
ANN	Artificial neural network
LP	Laser power
PF	Pulse frequency
SS	Scan speed
LS	Line spacing
D	Depth of ablation
W	Width of ablation
Ra	Surface roughness
GA-ELMSS	ELMSS optimized by genetic algorithm
GA-ELMPS	ELMPS optimized by genetic algorithm

## Appendix A

**Table A1:** Results of the technological test.

No.	LP/(W)	PF/(kHz)	SS/(mm/s)	D/(μm)	W/(μm)	MRR/(mm^3^/min)	LS/(μm)	Ra/(μm)
1	10	40	100	4.710	36.320	1.105	-	-
2	10	40	150	3.504	35.470	1.166	-	-
3	10	40	200	2.710	32.544	1.391	-	-
4	10	40	250	2.040	31.157	1.457	-	-
5	10	50	100	5.928	37.769	1.149	5	27.794
							10	23.326
							15	18.695
6	10	50	150	4.841	36.310	1.268	5	14.293
							10	11.015
							15	10.664
7	10	50	200	3.700	33.166	1.529	5	11.764
							10	9.155
							15	6.584
8	10	50	250	3.170	32.032	1.885	-	-
9	10	60	100	8.365	38.769	1.291	5	19.704
							10	16.624
							15	12.283
10	10	60	200	5.044	33.660	1.829	5	12.804
							10	10.180
							15	8.278
11	10	60	250	4.044	32.320	1.997	-	-
12	10	70	100	9.453	40.204	1.776	5	14.742
							10	12.070
							15	10.263
13	10	70	150	7.580	39.810	2.191	5	14.869
							10	13.097
							15	12.301
14	10	70	200	5.624	38.395	2.582	5	11.748
							10	7.905
							15	4.838
15	10	70	250	4.243	33.837	2.608	-	-
16	14	40	100	7.476	40.408	1.975	-	-
17	14	40	150	6.130	38.708	1.916	-	-
18	14	40	200	4.288	35.245	1.914	-	-
19	14	40	250	3.052	32.096	1.573	-	-
20	14	50	100	8.146	41.272	2.184	5	32.925
							10	28.327
							15	23.591
21	14	50	150	6.348	39.048	2.039	5	19.979
							10	15.159
							15	12.959
22	14	50	200	5.549	36.511	2.066	5	13.526
							10	11.306
							15	8.105
23	14	50	250	4.746	33.123	1.794	-	-
24	14	60	100	10.268	42.599	2.629	5	21.251
							10	18.972
							15	14.757
25	14	60	150	9.102	40.871	2.474	5	17.993
							10	15.614
							15	12.274
26	14	60	200	8.812	37.007	2.500	5	13.877
							10	11.260
							15	8.337
27	14	60	250	7.024	34.157	2.341	-	-
28	14	70	100	17.312	43.919	3.184	5	17.849
							10	13.181
							15	11.067
29	14	70	200	10.878	39.783	3.062	5	15.051
							10	14.290
							15	12.107
30	14	70	250	7.965	35.157	2.669	-	-
31	18	40	100	14.575	46.128	2.603	-	-
32	18	40	150	9.374	43.166	2.333	-	-
33	18	40	200	7.041	41.972	2.258	-	-
34	18	40	250	5.231	37.051	2.333	-	-
35	18	50	100	20.385	49.208	3.802	5	35.821
							10	30.670
							15	25.005
36	18	50	150	12.270	45.340	3.043	5	22.010
							10	19.117
							15	14.139
37	18	50	200	8.811	40.191	2.674	5	14.345
							10	10.596
							15	9.437
38	18	50	250	6.703	38.369	2.668	-	-
39	18	60	100	25.609	51.713	4.905	5	29.545
							10	22.808
							15	19.132
40	18	60	150	14.685	47.270	3.649	5	19.415
							10	17.802
							15	13.063
41	18	60	250	8.321	39.969	3.108	-	-
42	18	70	100	33.557	58.532	5.783	5	20.487
							10	18.945
							15	15.735
43	18	70	150	17.000	52.575	4.280	5	19.233
							10	15.224
							15	13.599
44	18	70	200	12.086	42.954	3.708	5	14.304
							10	10.192
							15	6.231
45	18	70	250	9.282	40.840	3.301	-	-
46	10	60	150	6.590	36.341	1.730	5	13.956
							10	11.522
							15	8.573
47	14	70	150	15.415	41.633	3.591	5	13.578
							10	8.079
							15	5.176
48	18	60	200	10.545	41.798	3.144	5	16.999
							10	14.704
							15	11.253

**Table A2:** The connection weights and thresholds of GA-ELMSS.

IW_1_	IW_2_	IW_3_	B	LW_1_	LW_2_	LW_3_
−0.232	−0.086	0.031	0.079	−1826.213	−4082.110	−1450.281
−0.036	0.481	−0.430	−0.080	−220.138	639.136	201.555
0.089	−0.113	−0.101	0.125	−754.249	−291.58	−908.682
0.432	−0.440	−0.442	−0.318	−245.316	299.979	106.916
0.172	−0.472	−0.481	0.408	472.233	−633.298	−283.131
0.274	−0.045	0.040	−0.303	630.142	−2520.600	−1486.372
−0.009	−0.293	0.339	0.087	−1177.209	2947.502	830.049
−0.182	0.265	0.287	0.158	−99.858	1920.822	1213.711
0.453	0.198	0.193	−0.302	3093.931	154.709	651.381
0.374	−0.185	−0.143	−0.395	−987.277	1859.971	1085.935
0.240	−0.489	0.032	−0.206	−51.833	438.757	−96.488
0.452	0.131	0.052	−0.227	−4406.521	−4427.502	−1876.558
0.463	0.105	0.115	−0.352	3389.136	2832.371	1202.500
−0.484	0.432	0.292	−0.205	−52.638	−57.240	158.769
−0.169	−0.348	−0.213	0.351	−15.892	−520.897	−243.700
−0.203	−0.067	−0.440	0.002	1529.101	−1179.513	−207.149
0.087	−0.031	0.457	0.396	1066.204	−1399.417	−492.390
0.235	−0.435	−0.292	−0.160	−3468.958	5082.055	1747.812
0.232	0.321	0.499	−0.435	386.744	−298.529	−139.302
−0.202	0.250	−0.022	−0.219	730.832	−362.843	−550.227
0.424	−0.442	−0.199	0.105	487.610	−1254.683	−173.397
0.419	0.433	−0.449	−0.047	8.212	43.563	23.585
−0.298	0.012	0.418	−0.119	−820.394	242.580	129.553
−0.087	0.384	0.308	0.138	−807.174	369.732	−260.524
0.292	−0.443	−0.308	−0.431	2302.590	−3053.327	−1091.668
0.014	−0.059	−0.435	0.336	−2022.303	974.542	−17.184
−0.344	0.227	−0.344	0.490	−330.872	306.562	90.751
−0.306	0.424	−0.235	−0.060	265.001	58.001	−103.546
0.412	−0.015	0.473	0.339	−67.134	35.861	74.545
0.112	0.357	−0.145	−0.063	−770.724	1100.53	153.023
−0.339	−0.163	−0.256	0.458	4189.418	395.267	695.384
0.302	−0.149	−0.289	0.401	228.638	313.967	661.793
−0.281	0.311	−0.056	−0.484	−791.866	−99.713	237.574

**Table A3:** The connection weights and thresholds of GA-ELMPS.

IW1	IW2	IW3	IW4	B	LW
0.004	0.329	−0.126	−0.178	−0.163	−284.652
−0.107	−0.283	0.479	−0.377	0.166	72.062
0.054	0.217	0.460	0.478	−0.063	223.608
0.268	−0.160	0.072	−0.436	−0.078	108.983
−0.077	−0.183	−0.328	−0.016	0.384	−765.715
0.282	−0.212	0.372	0.265	0.473	101.259
0.141	0.114	−0.388	0.483	−0.047	−29.873
−0.332	−0.045	0.000	−0.101	−0.357	−206.184
−0.130	−0.093	0.449	−0.024	−0.444	205.914
−0.478	−0.421	0.016	0.458	−0.359	86.762
−0.136	0.419	−0.296	0.338	0.300	−39.310
−0.438	−0.444	0.060	−0.464	0.315	4.860
0.065	0.229	0.036	−0.385	−0.140	829.225
−0.221	−0.239	−0.309	0.249	0.352	104.030
−0.498	−0.139	−0.026	0.086	-0.185	14.744
0.296	0.142	−0.451	−0.393	0.294	−100.834
0.040	−0.401	−0.364	0.462	0.283	65.728
0.009	−0.332	0.089	−0.316	−0.161	−29.440
0.447	−0.344	−0.036	0.423	−0.430	−13.284
−0.300	−0.242	0.151	-0.168	0.258	286.638
0.159	−0.120	−0.320	−0.184	-0.491	−179.574
−0.126	0.346	−0.370	−0.334	-0.330	124.116
0.482	−0.279	−0.428	−0.458	0.363	−42.063
−0.114	−0.170	−0.264	−0.353	0.090	353.406
−0.115	−0.424	0.141	0.400	0.472	173.414
0.375	0.412	−0.382	0.357	−0.377	15.628
−0.059	0.418	0.472	0.413	0.349	−13.191
−0.455	0.037	0.043	−0.326	0.170	−91.091
0.489	0.406	0.122	−0.114	0.374	−2.451
−0.198	−0.154	0.344	0.417	0.443	−96.488
0.408	0.394	0.495	0.015	−0.361	−22.932
0.223	0.000	−0.036	−0.443	−0.132	−87.396
0.335	−0.011	−0.457	−0.438	0.167	17.290
−0.156	−0.367	0.013	0.121	0.053	−274.688
−0.385	0.389	−0.056	0.336	0.038	92.504
0.003	0.050	0.352	0.114	-0.316	−976.839
−0.118	−0.468	−0.442	−0.493	−0.184	16.016
−0.006	−0.369	0.065	−0.355	−0.455	−29.808
−0.360	0.100	−0.079	0.470	0.385	59.787
0.469	−0.158	−0.029	0.290	0.430	8.016
−0.397	−0.284	−0.463	0.335	−0.451	−5.219
0.299	−0.187	−0.302	−0.258	0.390	430.767
0.230	−0.247	−0.348	−0.330	−0.481	52.736
−0.449	−0.335	−0.095	−0.291	−0.252	−28.102
0.242	0.335	−0.153	0.148	-0.499	382.970
0.200	0.195	−0.296	0.399	0.223	315.188
0.263	−0.169	−0.306	−0.164	0.433	−388.675
−0.087	−0.205	0.009	0.383	−0.149	768.795
−0.130	−0.061	0.041	−0.144	−0.242	−344.754
−0.122	−0.457	−0.013	0.079	0.500	209.373
0.058	−0.459	0.412	0.381	−0.076	−28.213
−0.429	−0.280	0.448	0.253	−0.443	−95.304
0.307	0.317	−0.284	0.274	−0.013	−160.962
−0.199	0.492	−0.056	0.221	0.103	−186.473
−0.492	−0.151	0.492	−0.442	0.206	−7.225
−0.264	−0.303	−0.027	0.469	−0.215	−216.882
0.240	−0.370	0.181	−0.164	0.197	−129.670
0.290	0.373	0.242	0.382	0.140	−24.274
0.458	0.018	−0.049	0.213	0.191	−308.920
0.139	−0.232	0.395	0.333	−0.406	59.406

## References

[1] Bonse J., Kirner S., Griepentrog M., Spaltmann D., Krüger J. (2018). Femtosecond Laser Texturing of Surfaces for Tribological Applications. Materials.

[2] Nsilani Kouediatouka A., Ma Q., Liu Q., Mawignon F.J., Rafique F., Dong G. (2022). Design Methodology and Application of Surface Texture: A Review. Coatings.

[3] Wu B., Deng L., Liu P., Zhang F., Duan J., Zeng X. (2017). Effects of picosecond laser repetition rate on ablation of Cr12MoV cold work mold steel. Appl. Surf. Sci..

[4] Rung S., Häcker N., Hellmann R. (2022). Micromachining of Alumina Using a High-Power Ultrashort-Pulsed Laser. Materials.

[5] Sun H., Li J., Liu M., Yang D., Li F. (2022). A Review of Effects of Femtosecond Laser Parameters on Metal Surface Properties. Coatings.

[6] Wang X., Duan J., Jiang M., Zhang F., Ke S., Wu B., Zeng X. (2017). Investigation of processing parameters for three-dimensional laser ablation based on Taguchi method. Int. J. Adv. Manuf. Technol..

[7] Mills B., Grant-Jacob J.A. (2021). Lasers that learn: The interface of laser machining and machine learning. IET Optoelectron..

[8] Chen Y., Wang H., Wu Y., Wang H. (2020). Predicting the Printability in Selective Laser Melting with a Supervised Machine Learning Method. Materials.

[9] Yousef B.F., Knopf G.K., Bordatchev E.V., Nikumb S.K. (2003). Neural network modeling and analysis of the material removal process during laser machining. Int. J. Adv. Manuf. Technol..

[10] Teixidor D., Grzenda M., Bustillo A., Ciurana J. (2015). Modeling pulsed laser micromachining of micro geometries using machine-learning techniques. J. Intell. Manuf..

[11] Jimin C., Jianhua Y., Shuai Z., Tiechuan Z., Dixin G. (2007). Parameter optimization of non-vertical laser cutting. Int. J. Adv. Manuf. Technol..

[12] Niu B., Chen J., Liu F. (2011). Optimization on the Fiber Laser Micro-Cutting of Thin Stainless Steel Sheet by Artificial Neural Networks. Adv. Sci. Lett..

[13] Dhupal D., Doloi B., Bhattacharyya B. (2007). Optimization of process parameters of Nd:YAG laser microgrooving of Al_2_TiO_5_ ceramic material by response surface methodology and artificial neural network algorithm. Proc. Inst. Mech. Eng. Part J. Eng. Manuf..

[14] Dixit S.R., Das S.R., Dhupal D. (2019). Parametric optimization of Nd:YAG laser microgrooving on aluminum oxide using integrated RSM-ANN-GA approach. J. Ind. Eng. Int..

[15] Ciurana J., Arias G., Ozel T. (2009). Neural Network Modeling and Particle Swarm Optimization (PSO) of Process Parameters in Pulsed Laser Micromachining of Hardened AISI H13 Steel. Mater. Manuf. Process..

[16] Dhara S.K., Kuar A.S., Mitra S. (2008). An artificial neural network approach on parametric optimization of laser micro-machining of die-steel. Int. J. Adv. Manuf. Technol..

[17] Yin Z., Liu Q., Sun P., Wang J. (2021). Study on Nanosecond Laser Ablation of 40Cr13 Die Steel Based on ANOVA and BP Neural Network. Appl. Sci..

[18] Dubey A.K., Yadava V. (2008). Laser beam machining—A review. Int. J. Mach. Tools Manuf..

[19] Huang G.-B., Zhu Q.-Y., Siew C.-K. Extreme learning machine: A new learning scheme of feedforward neural networks. Proceedings of the 2004 IEEE International Joint Conference on Neural Networks (IEEE Cat. No.04CH37541).

[20] Huang G.B., Zhu Q.Y., Siew C.K. (2006). Extreme learning machine: Theory and applications. Neurocomputing.

[21] Huang G., Song S., Gupta J.N.D., Wu C. (2014). Semi-Supervised and Unsupervised Extreme Learning Machines. IEEE Trans. Cybern..

[22] Zhu Q.Y., Qin A., Suganthan P., Huang G.B. (2005). Evolutionary extreme learning machine. Pattern Recognit..

